# Recanalization of thrombosed aneurysmal hemodialysis arterovenous fistulas using a hybrid technique based on data from a single center

**DOI:** 10.1186/s12882-022-02820-9

**Published:** 2022-05-14

**Authors:** Wei Liu, Meng Wu, Xu Wang, Xiao-kang Huang, Wen-jiao Cai, Teng-yun Ding, Liang-liang Duan, Rui Qiao, Yong-gui Wu

**Affiliations:** 1 Department of Nephropathy, The First Affiliated Hospital of Anhui Medical University, Hefei, Anhui 230022 P.R. China; 2 Department of Nephropathy, Anqing Municipal Hospital, Anqing, Anhui 246000 P.R. China; 3 Department of Ultrasonography, Anqing Municipal Hospital, Anqing, Anhui 246000 P.R. China; 4 Department of Cardiology, Anqing Municipal Hospital, Anqing, Anhui 246000 P.R. China; 5 Center for Scientific Research, Anhui Medical University, Hefei, Anhui 230022 P.R. China

**Keywords:** Thrombosis, Aneurysm, Arteriovenous fistula, Ultrasonography, Angioplasty

## Abstract

**Objective:**

To explore the technical specifications and clinical outcomes of thrombosed aneurysmal haemodialysis arteriovenous fistula (AVF) treated with ultrasound-guided percutaneous transluminal angioplasty combined with minimal aneurysmotomy.

**Methods:**

This case series study included 11 patients who had thrombosed aneurysmal AVF and underwent salvage procedures over a 13-month period. All procedures were performed under duplex guidance. Minimal aneurysmotomy was performed, along with manual thrombectomy and thrombolytic agent infusion, followed by angioplasty to macerate the thrombus and sufficiently dilate potential stenoses. A successful procedure was defined as immediate restoration of flow through the AVF.

**Results:**

The 11 patients (four males and seven females) had a mean age of 49.6 years ± 11.9 years. Six patients (54.5%) had two or more aneurysms. The mean aneurysm maximal diameter was 21.5 mm (standard deviation: ± 5.0 mm), and the mean thrombus length was 12.9 cm (8–22 cm). Ten (83.3%) of the 12 procedures were technically successful. The mean duration of operation was 150.9 minutes (standard deviation: ± 34.2 minutes), and mean postoperative AVF blood flow was 728.6 ml/min (standard deviation: ± 53.7 mi/min). The resumption of hemodialysis was successful in all 11 cases, with a clinical success rate of 100%. The primary patency rates were 90.0% and 75.0% at three and four months over a mean follow-up time of 6.3 months (3–12 months). The secondary patency rates were 90.4% at three and four months.

**Conclusion:**

A hybrid approach combining ultrasound-guided percutaneous transluminal angioplasty and minimal aneurysmotomy might be a safe and effective method for thrombosed aneurysmal AVF salvage.

## Introduction

The quality of haemodialysis is determined by the reliability and integrity of the haemodialysis vascular access. In terms of primary vascular access, a autologeous arteriovenous fistula (AVF) is considered the preferred choice because of greater reliability, less infections, fewer hospitalizations, lower mortality, and high cost-efficiency [1, 2]. However, the long term patency is usually good, but maturation failure is high, and thrombosis and vascular stenosis can lead to AVF failure in patients. [3].Another common complication of vascular access is arteriovenous (AV) access aneurysm, with incidences ranging from 5% to 60% in clinical studies [4, 5]. An outflow stenosis results in elevated intra-luminal pressure, recurrent canulations, and genetic factors that induce aneurysm formation [6].

Arteriovenous (AV) access aneurysms are inclined to cause AV access thrombosis, making AV access thrombectomy difficult. Surgical procedures have been widely used in the remodeling of AV access aneurysms, with the most prevalent methods being reconstruction by vasculectomy of partial aneurysm and replacement with artificial blood vessels [7]. Nonetheless, surgical procedures are more invasive, making it technically difficult to manage several concurrent stenoses and aneurysms positioned at various sections of the fistula. Surgical procedures can also reduce the number of canulation sites and increase the usage of dialysis catheters during the perioperative period. Endovascular procedures are increasingly being used to recanalization a thrombosed AV access [8, 9]. However, recanalization becomes difficult when an AVF is obstructed by oversized thrombi inside an aneurysm because of the expanded vascular space and old mural thrombus that may result in symptomatic pulmonary embolism.

Both surgery and endovascular procedures have several shortcomings, which may be mitigated or restrained by hybrid procedures such as a mixture of surgery and endovascular techniques [10]. We have now applied the same principles in a modified manner to an aggressive approach for salvaging thrombosed aneurysmal fistulas endovascularly using duplex scanning as the exclusive imaging modality combined with minimally invasive aneurysmotomy.

## Methods

### Study cohort

**Fig. 1:**
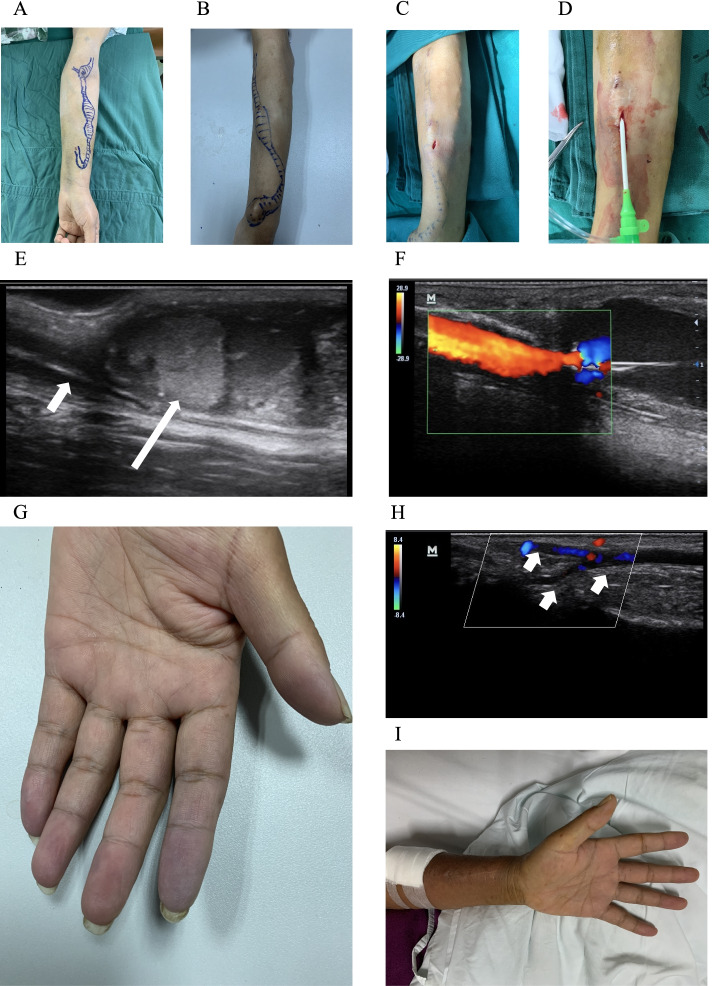
**(A** and **B)** Typical thrombosed aneurysmal fistulas. Body surface markings indicate the size of aneurysms and the range of thrombus. (**C**) The aneurysm wall was exposed using minimal aneurysmotomy. (**D**) An introducer sheath was inserted antegradely into the incision site under ultrasound guidance. (**E**) Aneurysm with massive organized thrombi (long white arrow) and proximal stenosis (short white arrow). (**F)** The color Doppler ultrasound blood flow signal was restored after the organizing thrombus in the aneurysm was removed and the stenosis was fully dilated. (**G**) The palm of same patient, three digits of the left hand were cyanotic. (**H**) A preoperative color Doppler ultrasound examination of palmar arch vessels of the patient indicated weak blood flow signal (short white arrow). (**I**) The cyanosis disappeared after thrombus removal

We reviewed the medical records retrospectively of all end-stage renal disease (ESRD) patients who had undergone a modified hybrid thrombectomy procedure of thrombosed aneurysmal fistula at the Anqing Municipal Hospital (a tertiary hospital affiliated to Anhui Medical University) between January 1, 2020 and January 31, 2021. The inclusion criteria were defined as follows: (I) on maintenance hemodialysis; (II) diagnosed with AV access true aneurysm; (III) represented the first time the AV access had thrombosed; and (IV) long segments of thrombosis from the anastomosis and the extent of the thrombus involved at least one entire aneurysm (Fig. 1A and 1B). Patients with artificial vascular graft thrombus and pseudoaneurysms were excluded.

### Modified hybrid thrombectomy procedure

Patients were referred to the vascular access team by the haemodialysis center if complications with AV access occurred during or after haemodialysis. Thromboses and aneurysms were confirmed using preoperative duplex ultrasound with a 6- to 14-MHz liner transducer, and the decision to begin the hybrid thrombectomy procedure was personalized based on the patient and strength of the judgement of the attending interventional nephrologist (WL). If a patient met the criteria for a modified hybrid thrombectomy, the procedure was performed within 24 hours of admission.

All surgeries were performed in a sterile operating room and no prophylactic antibiotic agents were used. The skin was sterilized using povidone iodine, and sterile protocols were strictly observed throughout the procedure. Because of the large number of thrombi, the initial treatment was a minimal aneurysmotomy. Aneurysmotomy sites were identified using ultrasound real-time monitoring at enlarged obstructed sections with the supreme diameters. After local anesthesia with 2–3 ml of 1% lignocaine, a skin incision of roughly 10 mm long was made lengthways on the enlarged fistula. The hypodermic tissue was dissected bluntly with a forcep to expose the blood vessel wall, and a lengthwise aneurysmotomy of approximately 8 mm was created (Fig. 1C). The clot was dislodged from the expanded section using with forceps and manual squeezing. We squeezed the thrombus in a specific sequence, first at the aneurysm, then in the proximal fistula, and finally between the anastomosis and the aneurysm.

The arterial anastomosis site was slightly compressed to prevent the thrombus from entering the feeding artery. If the thrombus crosses the anastomosis and becomes entangled in the artery, the anastomosis cannot be squeezed to prevent the thrombus from sliding off and causing embolization of the distal artery. The existence of any residual thrombi was detected using ultrasonography. A second incision was made when the clot in another expanded section could not be removed by manual squeezing. An introducer sheath (5–6 French) was inserted antegradely and retrogradely under ultrasound guidance via the incision site (Fig. 1D), through which a guide wire was inserted and a 5–7-mm balloon was passed to the residual thrombus area. The thrombus area was evenly injected with 2000 U of heparin and 200,000 U of urokinase via the balloon catheter, while the proximal return vein was appropriately blocked by a tourniquet during thrombolysis. After 10–20 minutes of thrombolysis, the balloon was reinserted to macerate the residual thrombus and dilate visible stenosis. The balloon was placed at the anastomotic site to block the blood flow after blood flow was recanalized. In addition, the vessel wall and skin layer were closed with a straightforward interrupted stitch. The obstructing balloon was withdrawn and recovery of the blood flow was confirmed. If severe stenosis persisted in the drain vessel, balloon angioplasty with a 5–7 mm balloon catheter was performed repeatedly. Angioplasty is usually performed until all stenotic sections are absolutely dilated (< 30% residual stenosis) and definite flow within the fistula is reconfirmed (Fig. 1E and 1F).

Fistula angiography was not routinely applied when the postoperative arm elevation sign indicated that the aneurysm could collapse and ultrasonography blood flow monitoring was standard. For haemodialysis, AVF can be punctured immediately after the procedure. Aspirin 100 mg was prescribed once daily for half a month when a small amount of mural thrombus that did not affect blood flow in the lumen persisted. Sutures were removed after a couple of weeks, and ultrasound scans were performed every three months. The protocol for this study was approved by the Anqing Municipal Hospital Institutional Review Board, and informed consent was waived due to its retrospective nature.

### Exposure variables

Patient-level variables included demographics (e.g., age, gender), renal failure etiologies (e.g., chronic glomerulonephritis, systemic lupus nephritis, and obstructive nephropathy), and comorbid conditions (e.g., smoking status, ischemic heart disease, and congestive heart failure). Characteristics of thrombosed aneurysmal fistulas included location of fistula, age of fistula, duration from onset of thrombus to intervention, aneurysm maximal diameter, thrombus length, symptoms, and history of previous interventions. Furthermore, variables related to the modified hybrid thrombectomy procedures were evaluated, including duration of operation, postoperative blood flow, potential stenosis, technical and clinical success rate, complications, follow-up patency, and types of intervention performed to preserve patency and to maintain functionality.

### Definitions

The success of thrombectomy and angioplasty procedures was determined based on the National Kidney Foundation Kidney/Dialysis Outcome Quality Initiative Guidelines (2019 updated) [11]. Clinical success was defined as the successful resumption of hemodialysis for at least one session. Technical success was defined as recanalization of hemodialysis access with 30% or less residual stenosis. Primary patency referred to the duration between the intervention and the next access thrombosis or repeat intervention. Secondary patency referred to the time between the intervention and access abandonment or surgically revision. Complications were categorized as major or minor according to the Society of Interventional Radiology (SIR) guidelines [12].

### Statistical analyses

For continuous variables, the data are summarized as mean, standard deviation, and range. For categorical variables, the data are summarized as count (percentage). A Kaplan-Meier analysis was used to calculate primary and secondary patency. All analyses were performed using the Statistical Package for the Social Sciences (SPSS) ver. 23 software (SPSS Inc., Chicago, IL, USA).

## Results

Between January 1, 2020 and January 31, 2021, 11 patients underwent 12 hybrid thrombectomy procedures. Patient basic characteristics are shown in Table 1. There were four males (36.4%) and seven females (63.6%), with a mean age of 49.6 years (standard deviation: ± 11.9 years). Etiologies of renal failure were significantly higher with chronic glomerulonephritis (eight patients, 72.7%), whereas lupus nephritis, obstructive nephropathy, and ANCA-associated vasculitis each accounted for 9.1% (one patient). The patient with lupus nephritis also had an abdominal aortic aneurysm.

**Table 1:** Basic characteristics of the patients

*Variable*^*a*^	*Patients (n=11)*
Gender	
Male	4 (36.4%)
Female	7 (63.6%)
Age (years)	49.6 ± 11.9
Etiologies of renal failure	
Chronic glomerulonephritis	8 (72.7%)
Systemic lupus nephritis	1 (9.1%)
Obstructive nephropathy	1 (9.1%)
ANCA-associated vasculitis (AAV)	1 (9.1%)
Comorbidities	
Abdominal aortic aneurysm	1 (9.1%)
Hyperthyroidism	1 (9.1%)
Gout	1 (9.1%)
Smoking	3 (27.3%)
Ischemic heart disease	3 (27.3%)
Congestive heart failure	3 (27.3%)

*ANCA* Antineutrophil cytoplasmic antibody

^a^Continuous data are shown as the mean ± standard deviation or the

mean (range) and categorical data as number (%)

Characteristics of thrombosed aneurysmal fistulas are shown in Table 2. All patients had forearm AV fistulas, with the major location of aneurysm located at perianastomotic AVF (five patients, 45.5%) and distal AVF (nine patients, 81.8%). Six patients (54.5%) had two or more aneurysms. The mean age of the fistula was 60.8 months, and the duration from onset of thrombus to intervention was 48–72 hours, with the exception of one patient who had a 432-hour delay. The mean aneurysm maximal diameter was 21.5 mm (standard deviation: ± 5.0 mm), and the mean thrombus length was 12.9 cm (8–22 cm). As shown in Table 2, all patients had local pain at the thrombosis site. One patient suffered severe pain as a result of the thrombus falling into the distal artery, and three digits of the left hand were cyanotic before surgery. A Doppler ultrasound examination of palmar arch vessels of this patient indicated a weak blood flow signal. The patient was treated with catheter-directed thrombolysis, and the pain on the digits and cyanosis were relieved after intervention (Fig. 1G, 1H and 1I). Three patients (27.3%) had a history of percutaneous transluminal angioplasty (PTA), three patients (27.3%) had a history of AVF surgical revision for non-thrombotic events, and one patient (9.1%) had a history of kidney transplantation.

**Table 2:** Characteristics of thrombosed aneurysmal fistulas

*Variable*^*a*^	*Patients(n=11)*
Location of fistula	
Left forearm	10 (90.9%)
Right forearm	1 (9.1%)
Age of fistula (months)	60.8 ± 34.9
Time from onset of thrombus toIntervention (hours)	87 (24–432)
Thrombus length (cm)	12.9 (8–12)
Location of aneurysm	
Perianastomotic AVF	5 (45.5%)
Mid AVF	1 (9.1%)
Distal AVF	9 (81.8%)
Two or more aneurysms	6 (54.5%)
Aneurysm maximal diameter(mm)	21.5 ± 5.0
Symptom	
Local pain	11 (100%)
Finger cyanosis	1 (9.1%)
History of previous intervention	
PTA	3 (27.3%)
AVF surgical revision	3 (27.3%)
Kidney transplantation	1 (9.1%)

*PTA* Percutaneous transluminal angioplasty, *AVF* Arteriovenous fistula

^a^Continuous data are shown as the mean ± standard deviation or the

mean (range) and categorical data as number (%)

The mean duration of the surgery was 150.9 minutes (standard deviation: ± 34.2 minutes), full restoration of the fistula blood flow was confirmed in all patients (100%), and the technical success rate was 83.3% (10 out of the 12 procedures). One technical failure occurred when an obstructed segment with severe stenosis could not be bridged with the guide wire, necessitating a surgical procedure. In the other failed case, the stenosis was not completely treated, resulting in postoperative rethrombosis, and salvage recanalization was successfully repeated. The mean postoperative AVF blood flow was 728.6 ml/min (standard deviation: ± 53.7 mi/min), all patients successfully resumed hemodialysis, and the clinical success rate was 100%. In all cases, intraoperative color Doppler ultrasound confirmed the existence of vascular stenosis. According to Per SIR definitions [12], three major and one minor complications occurred in two patients (18.2%). The three major complications were post-procedural rethrombosis, fistula rupture, and incision infection. Unfortunately, these major complications occurred in one patient. Cultures of the infected skin confirmed the presence of *Staphylococcus epidermidis,* which was the causative organism, and sensitive antibiotic regimens were administered. One week after the infection, the fistula ruptured during a dressing change, necessitating an urgent open surgical procedure to repair the breach. One minor complication was mild postoperative forearm edema that did not required any intervention.

**Fig. 2:**
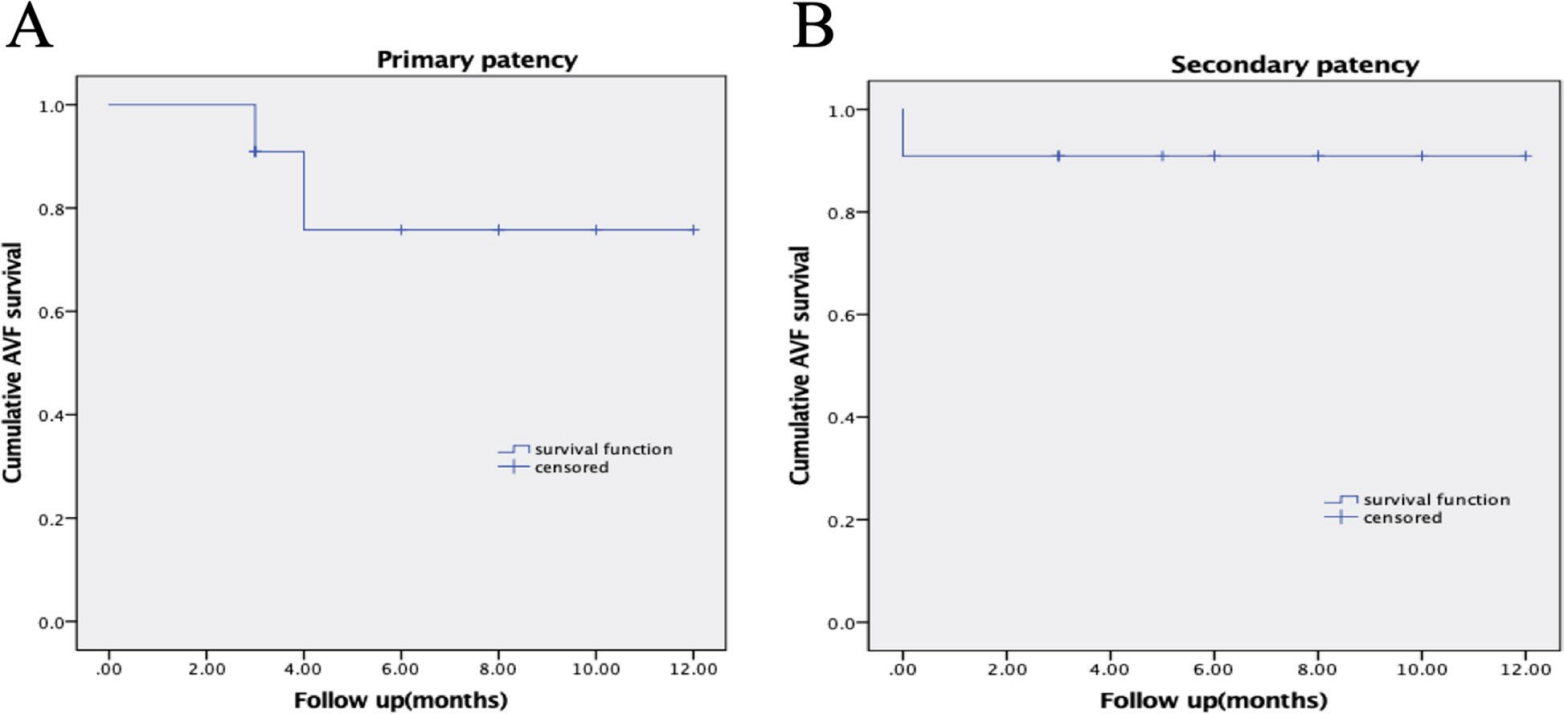
Kaplan-Meier survival curves for AVF primary patency rates (**A**) and secondary patency rates (**B**) after restoration of blood flow

The mean follow-up duration was 6.3 months (3–12 months). Primary patency rates were 90.0% after three months and 75.0% after four months. Secondary patency rates were 90.4% after three months and 90.4% after four months (Figure 2). During the follow-up period, two patients underwent two repeat interventions to maintain AVF patency. One patient underwent repeated modified hybird procedure during a 4-month follow-up period because of rethrombosis. Another patient underwent repeated PTA for decreased fistula flow, which rendered effective dialysis impossible.

## Discussion

The incidence and prevalence of AV access aneurysms are not clearly defined, partially due to the inaccurate and inconsistent criteria. Balaz and Bjorck [4] defined an AVF aneurysm as a dilation of all three vascular layers with a diameter of > 18 mm or roughly three times the diameter of the outflow vein of a mature AVF, we adopted their definition of AVF aneurysm in our study. There are also other classifications due to the lack of consensus [13]. Aneurysms can form anywhere along the AV access, including the inflow artery [14], but they are commonly located in the outflow vein. KDOQI guidelines 2019 suggested AV access aneurysms be divided into 6 types based on their locations and numbers [11]. The hemodynamic changes induced by AV access creation causes AV access dilatation. Hemodynamic alterations are possibly aggravated by recurring canulations and vascular wall damage, and they can lead to aneurysms when combined with high intra-luminal pressures from any outflow stenosis. Aneurysms can induce AV access thrombosis, which is the leading cause of AVF disfunction. Thrombosis is also one of the reasons for discarding an aneurysmal AVF [7].

Access thrombosis can be managed using surgical procedures and endovascular technology, although both have certain shortcomings for aneurysmal AV accesses with thrombi. There are no randomized controlled trials comparing endovascular with surgery procedures for thrombosed aneurysmal fistulas, and the quality of the pertinent evidence to guide therapy is poor. According to KDOQI, open surgical procedure should be considered the standard treatment for AV access aneurysms, with the specific approach determined based on native expertise (Expert Opinion). The procedure may include a scheme for staged restoration of various aneurysms to avoid implanting a central venous catheter during the perioperative period [11]. However, there are no recommendations for thrombosed aneurysmal fistulas.

Ahn [15] reported on sixteen patients who had endovascular recanalization of a thrombosed autologeous AVF combined with an aneurysm. Mechanical thrombectomy was used for recanalization, followed by additional treatments. Balloon angioplasty was used for all stenoses in 15 patients (93.8%), and stents were inserted in two patients (12.5%) because of central vein stenoses. All patients had outflow draining vein stenoses, five patients (31.8%) had AV anastomosis and juxta-anastomosis stenoses, and three patients (18.8%) had central vein stenoses. The primary patency rates at three, six, and twelve months were 70.5%, 54.8%, and 31.3%, respectively, whereas the secondary patency rates were 70.5%, 70.5%, and 47.0%, respectively. In this study, the 12-month primary patency rate was relatively low, whereas the secondary patency rate increased to 47%. However, if recanalization was successful, subsequent interventional treatment can often prolong the lifetime of a aneurysmal AVF. In our study, We used duplex scanning as a preoperative examination method and intraoperative real-time monitor, while they were guided by fistulography. The aneurysmal section was not used as a canulation site, although we took advantage of aneurysms as an endovascular intervention puncture site. Additionally, the type of AVF was mainly upper-arm brachiocephalic (87.5%), as opposed to our study (100% forearm AVF), and the detailed information of aneurysms and thrombus, particularly the measurement data of the color Doppler ultrasound is not mentioned.

Recently, “Stent tunnel technique”, which is a novel endovascular treatment technique of inserting nitinol auto-expandable uncovered stents stretching through the whole puncture site area, thereby creating a tunnel inside the thrombus, was reported to salvage thrombosed autologeous AVF with extensive aneurysm [16]. They reported good patency rates,but the results could not be further interpreted due to the short follow-up and incomplete vascular access characteristics. Zink et al [17] reported a 29% complication rate with the use of stent grafts for salvaging AV access, which included aneurysms/pseudoaneurysms and specific complications such as migration, fracture, erosion, and rupture.

Lambert [18] compared open surgical and radiological interventions for thrombosed arteriovenous access and found that interventional radiological thrombectomies had a lower primary failure rate and better assisted primary patency than surgical thombectomies. However, interventional radiological thrombectomies had a lower intervention-free survival rate and required additional procedures to maintain patency. Hybrid procedures were introduced in the treatment of AV access with aneurysm in previous studies [10, 19]. The treatment consists of the open and endovascular steps, which are performed separately. Hybrid procedures are less invasive compared to open surgery, allowing for multi-site treatment of various concurrent stenoses and aneurysms located in diverse sections of the fistula, as well as the preservation of an extended section of dialysis fistula accessible for canulation.

Joo SM [20] reported a novel technique of minimally invasive approach in the recanalization of thrombosed aneurysmal AVF, in which a small incision was made on the enlarged aneurysm, and thrombi were removed with forceps and Fogarty catheters via the incision site. A balloon catheter was inserted through the incision site to perform balloon angioplasty when significant stenosis was found. The duration of follow-up period ranged from 2 to 32 months (mean: 12 months; median: 10.5 months). Primary patency rates were 92% after three months, 68% after six months, and 19% after 12 months. Secondary patency rates were 100% after three months, 100% after six months, and 92% at 12 months. High technical success and secondary patency rates were achieved in these patients..

Our study has unique characteristics compared with the previous studies. First, our patients had more severe thrombotic volume, with one patient experiencing a thromboembolism emergency. The mean aneurysm maximal diameter was 21.5 mm (standard deviation: ± 5.0 mm) and the mean thrombus length was 12.9 cm (8–22 cm). One patient had thrombosis for 18 days (432 hours) and was successfully recanalized. In our experience, the time of thrombus formation did not seem to be an absolute contraindication for endovascular procedures, and most of the patients could be recanalized provided that calcification thrombosis is not combined.

Second, all of our patients had forearm AV fistulas, which could be explained by the low proportion of upper arm fistulas in our hospital. If the forearm AV fistulas failed, a prosthetic vascular graft in the forearm was preferred.

Third, duplex scanning was the exclusive imaging modality during the procedure. The Doppler color ultrasound allows for a more precise visual image of the thrombus than radioscopy, aiding in the prevention of blood vessel embolism when working in the anastomotic space. Ultrasound decreases radiation exposure for the patient and, most importantly, for the personnel by allowing for shorter durations for radioscopy. Although sonography cannot provide the same panoramic image of the fistula as radioscopy, we believe that a comprehensive and careful evaluation of preoperative color Doppler Ultrasound and skilled ultrasound techniques can compensate for this flaw.

Fourth, three major complications occurred in the same patient, including post-procedural thrombosis and fistula rupture secondary to incision infection. Repeated incisions at the same aneurysm site, as well as prolonged operation time, may have caused incision infection. The immediate postoperative rethrombosis was due to the negligence in evaluating the stenosis during the procedure. The degree to which the stenotic segments were dilated will also have an important impact on the long-term patency rate of the aneurysmal fistulas. In this group of cases, one patient had rethrombosis in the fourth month of follow-up, and another had decreased fistula blood flow in the third month; re-intervention was basically linked to the restenosis.

### Limitations

Our study has a couple of limitations. First, it was a retrospective study, which suggests that certain events may have been missed throughout the follow-up period. Second, our research only included a small number of patients, and the study period was brief. Despite these limitations, our technical success rate, short-term primary patency, and secondary patency were comparable to those reported in previous studies. Nonetheless, the sample size should be increased in future research and continue with collection of follow-up data in order to analyze long-term patency rates. Finally, because this was not comparative study, a comparison between surgical strategies and our approach is required in the future.

## Conclusions

In conclusion, ultrasound-guided percutaneous transluminal angioplasty combined with minimal aneurysmotomy is potentially a safe and effective approach for the salvage of thrombosed aneurysmal AVF.

## Data Availability

All data generated or analysed during this study are included in this published article.

## Abbreviations

AVF: Arteriovenous fistula
AV: Arteriovenous
ESRD: End-stage renal disease
SIR: Society of Interventional Radiology
KDOQI: Kidney Disease Outcomes Quality Initiative
PTA: Percutaneous transluminal angioplasty
ANCA: Antineutrophil cytoplasmic antibody
AAV: ANCA-associated vasculitis
